# Genetically induced dysfunctions of Kir2.1 channels: implications for short QT3 syndrome and autism–epilepsy phenotype

**DOI:** 10.1093/hmg/ddu201

**Published:** 2014-05-02

**Authors:** Elena Ambrosini, Federico Sicca, Maria S. Brignone, Maria C. D'Adamo, Carlo Napolitano, Ilenio Servettini, Francesca Moro, Yanfei Ruan, Luca Guglielmi, Stefania Pieroni, Giuseppe Servillo, Angela Lanciotti, Giulia Valvo, Luigi Catacuzzeno, Fabio Franciolini, Paola Molinari, Maria Marchese, Alessandro Grottesi, Renzo Guerrini, Filippo M. Santorelli, Silvia Priori, Mauro Pessia

**Affiliations:** 1 Department of Cell Biology and Neuroscience, Istituto Superiore di Sanità, Viale Regina Elena 299, Rome 00161, Italy,; 2 Clinical Neurophysiology Laboratory, Department of Developmental Neuroscience and; 3 Molecular Medicine Laboratory, IRCCS Stella Maris Foundation, Pisa, Italy,; 4 Faculty of Medicine, Section of Physiology & Biochemistry, Department of Experimental Medicine,; 5 Faculty of Medicine, Department of Experimental Medicine and; 6 Department of Chemistry, Biology and Biotechnology, University of Perugia, Perugia, Italy,; 7 Molecular Cardiology, IRCCS Salvatore Maugeri Foundation, Pavia, Italy,; 8 Department of Pharmacology, Istituto Superiore di Sanità, Rome, Italy,; 9 Computational Medicine and Biology Group, CASPUR, Rome, Italy and; 10 Pediatric Neurology Unit and Laboratories, Children's Hospital A. Meyer-University of Florence, Florence, Italy

## Abstract

Short QT3 syndrome (SQT3S) is a cardiac disorder characterized by a high risk of mortality and associated with mutations in Kir2.1 (*KCNJ2*) channels. The molecular mechanisms leading to channel dysfunction, cardiac rhythm disturbances and neurodevelopmental disorders, potentially associated with SQT3S, remain incompletely understood. Here, we report on monozygotic twins displaying a short QT interval on electrocardiogram recordings and autism–epilepsy phenotype. Genetic screening identified a novel *KCNJ2* variant in Kir2.1 that (i) enhanced the channel's surface expression and stability at the plasma membrane, (ii) reduced protein ubiquitylation and degradation, (iii) altered protein compartmentalization in lipid rafts by targeting more channels to cholesterol-poor domains and (iv) reduced interactions with caveolin 2. Importantly, our study reveals novel physiological mechanisms concerning wild-type Kir2.1 channel processing by the cell, such as binding to both caveolin 1 and 2, protein degradation through the ubiquitin–proteasome pathway; in addition, it uncovers a potential multifunctional site that controls Kir2.1 surface expression, protein half-life and partitioning to lipid rafts. The reported mechanisms emerge as crucial also for proper astrocyte function, suggesting the need for a neuropsychiatric evaluation in patients with SQT3S and offering new opportunities for disease management.

## INTRODUCTION

Mutations in the *KCNJ2* gene, encoding the inwardly rectifying K^+^ channel Kir2.1, are responsible for the rare Andersen-Tawil syndrome (OMIM 170390), a condition characterized by periodic paralysis, cardiac arrhythmia and skeletal abnormalities (1). Affected patients also display a distinct neurocognitive phenotype characterized by deficits in executive function and abstract reasoning (2). The disease is linked to a loss of function of Kir2.1 channels (3). Individuals harboring mutations in *KCNJ2* may also present mood disorders and seizures (4–6). Notably, seizure susceptibility associated with cardiac arrhythmia have been described in several *K^+^ channelepsies* that may increase the risk to sudden unexpected death in affected patients (7).

SQT3s (OMIM 609622) is another cardiac disorder characterized by QT shortening, ventricular tachyarrhythmias and atrial fibrillation that is caused by *gain-of-function* mutations in *KCNJ2* (8–10). The electrophysiological alterations that accompany SQT3S have been investigated in details demonstrating that *gain-of-function* mutations in Kir2.1 caused an increase in the amplitude of either the inward-current (such as for the D172N variant) or outward-current (such as for the E299V and M301K changes). To date, neither the molecular mechanisms leading to channel dysfunction nor the potential consequence on other organs expressing the channel, including the brain, are known.

We recently reported on two homozygous twins manifesting intellectual disability, autism spectrum disorder (ASD), and a history of infantile spasms where we detected *gain-of-function* mutations in *KCNJ10*, encoding the Kir4.1 channel (11). Those findings highlighted an emerging role for the inwardly rectifying K^+^ channels dysfunction in autism–epilepsy associated with intellectual disability, which warranted further investigations (11,12). We herein report on the identification of a new p.K346T mutation in *KCNJ2* in *cis* with the previously detected p.R18Q variant in *KCNJ10* (11). The pathogenic relevance of the mutation was assessed in *Xenopus laevis* oocytes, HEK293 and glial-like cells. We demonstrated that the K346T mutation causes *gain of function* of the Kir2.1 channels by altering their trafficking and stabilization and suggest that the novel *KCNJ2* variant has a combined effect on cardiac rhythm and neuropsychiatric phenotype.

## RESULTS

### Identification of a new KCNJ2 mutation in homozygous twins exhibiting SQT3S and autism–epilepsy phenotype

**Figure 1.**
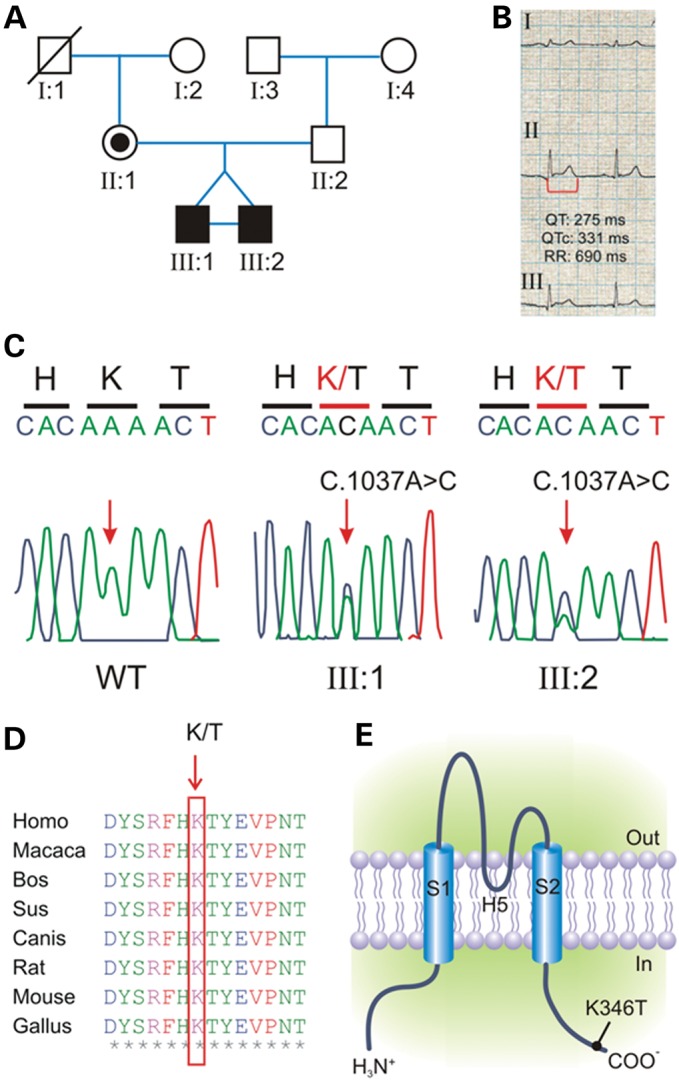
Mutation detection by sequence analysis of the KCNJ2 coding region. (**A**) Pedigree of the family harboring a novel mutations in *KCNJ2*. Squares are males and circles females; solid black symbols represent propositi and slash deceased individual. (**B**) ECG recording from propositi showing shortened QT interval (heart rate, 87 bpm; QT 275 ms; paper speed 25 mm/s). (**C**) Electropherograms showing the heterozygous c.1037A>C transition (arrow-headed), predicting a novel non-synonymous p.K346T variant in propositi compared with the sequence of a healthy individual (WT). (**D**) Alignments of several *KCNJ2* sequences flanking the K346T substitution (K/T, arrow-headed) showing that this residue is highly conserved in several vertebrate species. (**E**) Schematic representation of the membrane topology of a human Kir2.1 subunit indicating the position of the p.K346T variant.

The clinical case of the two probands has been reported both as SI data and elsewhere (11). In brief, two 9-year-old identical twins (Fig. 1A) displayed epilepsy and severe impairment of social interaction and communication, associated with stereotypes and repetitive behaviors, which were consistent with DSM-IV-TR criteria for ASD. Both children showed an electrocardiogram (ECG) with a markedly short repolarization time and conspicuously narrow and peaked T waves (QTc interval, 331 ms) (Fig. 1B). A novel heterozygous *KCNJ2* variant (c.1037A>C, p.K346T) was identified, by direct gene sequencing (Fig. 1C). The mutation was also found in the mother but it was absent in 400 ethnically matched control chromosomes (Fig. 1A and C) and was not found in large SNP databases (dbSNP and eversusgs.washington.edu/EVS/). Multiple sequence alignment showed that the lysine residue at position 346 (K346) is highly conserved in several vertebrate species (Fig. 1D) and lies in the cytoplasmic C-terminus domains of Kir2.1 channel (Fig. 1E).

### Functional characterization of homo- and heteromeric channels harboring the K346T mutation

**Figure 2.**
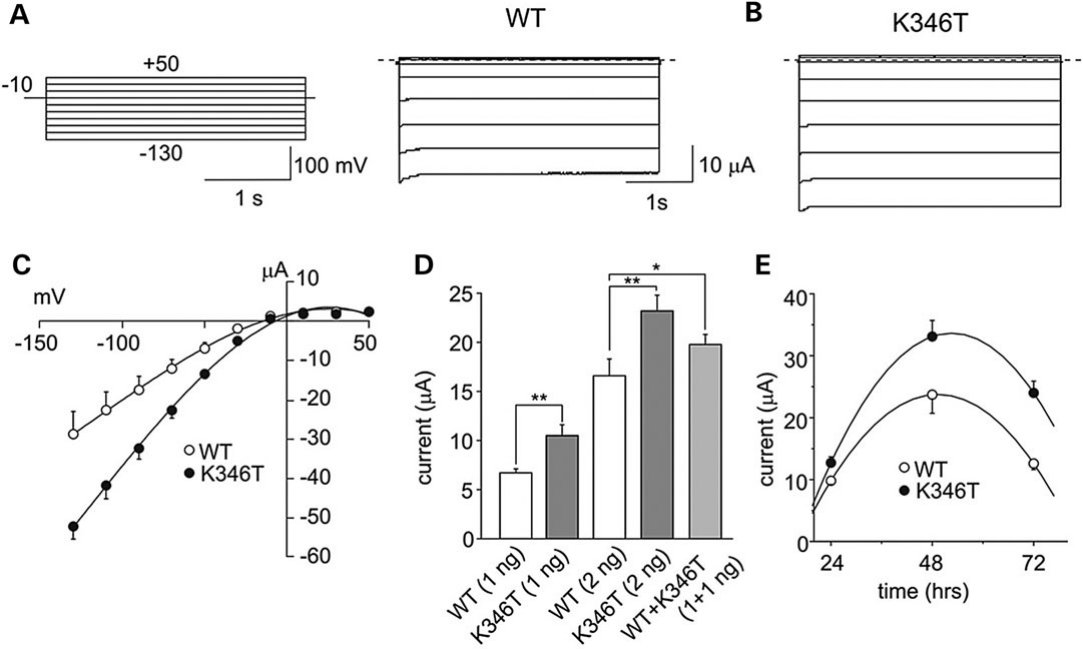
The K346T mutation increases Kir2.1 currents. Sample current families recorded from oocytes expressing WT (**A**) or K346T (**B**) mRNA (the pulse protocol is shown as inset). (**C**) I–V relationships for WT (white circles, 3 ng) and K346T (black circles, 3 ng) channels. (**D**) Current amplitudes recorded at −100 mV from cells injected with the indicated mRNAs whose amounts are reported in brackets (mean ± SEM; *n* = 120; *P < 0.05; **P < 0.01). (**E**) Current amplitudes recorded at −60 mV for WT (white circles) and K346T (black circles) channels and plotted as a function of time after mRNA injection (mean ± SEM; *n* = 6).

PolyPhen-2 software analysis used to predict the impact of the protein sequence variant p.K346T (13) suggested that p.K346T was a highly damaging mutation with a score of 0.918 (range: 0.1 low–1 high probability of severe variant). These findings prompted us to further investigate the pathogenic relevance of the p.K346T mutation by two-electrode voltage-clamp (TEVC) recording of K^+^ currents from *X. laevis* oocytes expressing either wild-type (WT) or K346T channels (Fig. 2). The expression of K346T channels yielded membrane currents, recorded in high extracellular K^+^ concentration (90 mm KCl), with macroscopic kinetics, inward-rectification and pH sensitivity (14) similar to WT (Fig. 2A and B; Supplementary Material). However, the I–V relationships for K346T channels had larger amplitudes than WT when equal amounts of the relevant mRNAs were expressed (Fig. 2C). Averaged current amplitudes linearly depended upon the amount of mRNA injected (Fig. 2D). To mimic the heterozygous state of the disease, WT and K346T mRNAs were co-injected at 1:1 ratio. Also this procedure yielded current amplitudes larger than the control (Fig. 2D). Moreover, the evaluation of the time course of K346T channel surface expression showed that the mutation resulted in faster raising and slower decaying phases than the WT (Fig. 2E). To compare these results with those obtained with the other KCNJ2 mutation associated with SQT3S, we expressed the Kir2.1 channels in HEK293 cells, and used a near-physiological K^+^ gradient. The gain-of-function effects assessed in HEK293 cells under these conditions (Supplementary Material) are similar to the D172N mutation that we originally described (8), suggesting that the p.K346T mutation results in increased Kir2.1 surface expression. However, larger macroscopic K^+^ currents may result from Kir2.1 single-channel conductance increase or channel-gating changes. To address this issue, single-channel current recordings were performed from *X. laevis* oocytes. Supplementary Material, Figure S1 shows representative recordings for WT (Supplementary Material, Fig. S1A) and K346T (Supplementary Material, Fig. S1B) obtained at −100 mV in the cell-attached configuration of the patch clamp. Event-by-event analysis revealed no significant differences in either unitary slope conductance (WT = 42.0 ± 1.4 pS; K346T 38.9 ± 1.0 pS; *n* = 6; *P* > 0.05) (Supplementary Material, Fig. S1C), rectification properties or obvious changes in gating parameters (I. Servettini, unpublished observation).

### The p.K346T mutation enhances membrane expression in astrocytoma cells

**Figure 3.**
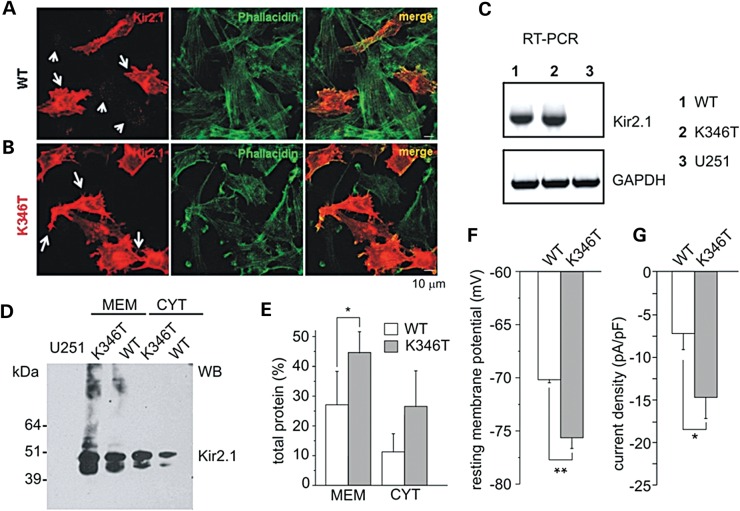
Characterization of astrocytoma cells expressing WT and K346T channels. Co-immunofluorescences of cells expressing WT (**A**) or K346T (**B**) channels with anti-Kir2.1 pAb (red) and FITC-conjugated phallacidin (green) show that WT channels are localized in perinuclear vesicles (short arrows in A) and occasionally at plasma membranes (long arrows in A), while mutated channels are mainly expressed at plasma membranes (long arrows in B). Scale bar: 10 μm. (**C**) RT-PCR analysis of Kir2.1 mRNA in WT (1), K346T (2) channel or empty-vector expressing U251 cell lines (3). GAPDH housekeeping gene normalizes the amount of template. (**D**) WB analysis of membrane (MEM) and cytosolic (CYT) proteins derived from WT or K346T Kir2.1-expressing cells after Histidine co-purification. Molecular weight markers are on the left (kDa). (**E**) Densitometric analysis of protein bands from four independent experiments (mean ± SEM, *P < 0.05). (**F**) The resting membrane potential and (**G**) current density (at −100 mV) were evaluated in cells expressing WT (white bars) or K346T (gray bars) channels (data are mean ± SEM; *n* = 6; *P < 0.05; **P < 0.01).

Kir2.1 channels are normally expressed in both cardiac myocytes and astrocytes (15–18). Thus, to explore whether the K346T mutation enlarges current amplitudes by increasing surface expression of the channel in an astrocyte-like cell context, we used U251MG cells stably expressing WT or K346T. To investigate WT and mutated Kir2.1 channels intracellular distribution in astrocytoma cells, we carried out immunofluorescence experiments and observed that WT channels were mostly localized in cytoplasmic vesicles distributed in perinuclear areas (Fig. 3A, short arrows) and, in ∼20–30% of the cells, also at plasma membrane level (Fig. 3A, long arrows). The prevalent intracellular localization of WT Kir2.1 channels in astrocytoma cells is consistent with previous findings obtained from rodent brain astrocytes (19). In contrast, the majority of cells (60–80%) expressing K346T mutant showed channels abundantly distributed along cell membranes, particularly at end-feet, filopodia-like structures and cell–cell contacts (Fig. 3B, long arrows), where Kir2.1 partially co-localizes with actin, and also at intracytoplasmic vesicles (Fig. 3B). RT-PCR analysis indicated that WT and K346T cells expressed comparable levels of recombinant gene mRNAs (Fig. 3C), suggesting no differences in the infection levels between the two cell populations. In the same amplification conditions, no Kir2.1 mRNA could be detected in mock-infected cells (Fig. 3C), confirming the undetectable expression of endogenous Kir2.1 (18). We corroborated the immunostaining differences with western blotting (WB) analysis (Fig. 3D) that showed K346T channels more abundantly expressed than WT proteins, particularly in the membrane-derived protein fractions (Fig. 3D and E). Patch-clamp recordings confirmed these data by revealing that the resting membrane potential of cells expressing the mutant channels was on average ∼6 mV more negative than the WT (Fig. 3F; Supplementary Material, Fig. S2), and the current densities were larger than the WT at both more positive and negative potentials than *E*_K_ (Fig. 3G; Supplementary Material, Fig. S2). These results altogether indicated that the p.K346T mutation exerted gain-of-function effects regardless of the expression system used.

### The K346T mutation increases protein stability in astrocytoma cells

**Figure 4.**
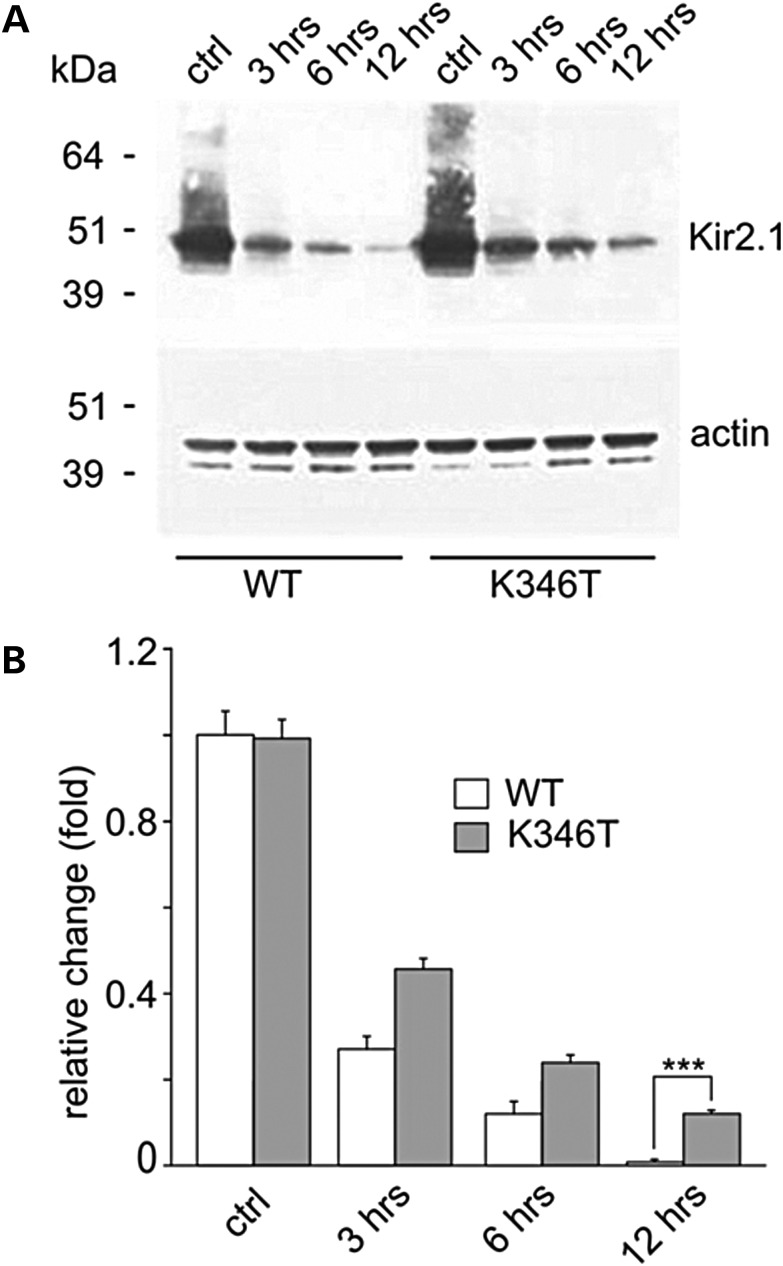
The K346T mutation increases protein stability. (**A**) WB analysis of protein extracts derived from cells expressing WT and K346T channels treated with the protein synthesis inhibitor cycloheximide for 3, 6 and 12 h. WT protein degradation is almost complete after 12 h treatment, while K346T protein is still detectable at this time. Actin is used as loading control. Molecular weight markers are on the left (kDa). (**B**) Densitometric analysis of protein bands normalized with respect to the amount of either WT (white bar) or K346T (gray bar) Kir2.1 protein in control conditions. Data are expressed as mean ± SEM from four independent experiments (***P < 0.001).

The slow time course of K346T current decay over several days after mRNA injection (see Fig. 2E), the enhancement of membrane expression and current density induced by K346T in the presence of normal mRNA expression (see above), raised the possibility that these effects could result from increased protein trafficking to and/or stabilization at the plasma membrane. To verify this possibility, cells expressing WT and K346T channels were treated for different periods—3, 6 and 12 h—with cycloheximide, a protein synthesis inhibitor (20). Subsequent WB analysis revealed that degradation of WT protein was faster than that of K346T, particularly after 12 h of cycloheximide treatment (Fig. 4A and B), suggesting that the p.K346T mutation results in greater protein stability.

To verify whether p.K346T mutation influenced Kir2.1 interactions with proteins known to modulate channel trafficking and/or plasma membrane stabilization (15,21,22), we used the His-affinity co-purification system and WB analysis as previously described (23,24). We tested syntrophin, α-dystrobrevin and Rac-1, without finding significant differences in the amount of co-purified proteins between WT and K346T expressing cells (Supplementary Material, Fig. S3). Aquaporin-4 and connexin-43 could not be detected among Kir2.1 interactors (M.S. Brignone, unpublished observations). In contrast, we found the co-presence of either Kir4.1 or Kir5.1 with Kir2.1 in the protein eluates derived from both WT- and K346T-expressing cells, although the mutation did not affect the possible interactions between these subunits (Supplementary Material, Fig. S3).

### K346T influences the ubiquitylation and proteasomal degradation of Kir2.1 channels

Ubiquitin (Ub) plays an essential role in the degradation of membrane proteins. Generally, the final step of the Ub-binding cascade creates an isopeptide bond between a lysine of the target protein and the C-terminal glycine of Ub. The involvement of a lysine residue in Kir2.1 stability and its distinct location in the cytoplasmic environment (see below Supplementary Material, Fig. S5) let us postulate that ubiquitylation could play a role in this process as Ub has been found to regulate surface expression and degradation of other members of the Kir family (25). Thus, we evaluated the background ubiquitylation levels of recombinant WT and K346T proteins by performing WB analysis with anti-polyubiquitin and anti-Kir2.1 antibodies and compared with that of K346T. Equal amounts of His-tagged WT and K346T protein eluates were resolved by SDS–PAGE and ubiquitylation levels were evaluated by WB (Supplementary Material, Fig. S4A). These experiments first revealed that Kir2.1 is ubiquitylated; they also showed that the ubiquitylation levels for K346T channels were lower than the WT (Supplementary Material, Fig. S4A and B). We confirmed that these data by using an *in vitro* ubiquitylation assay. Cells expressing WT or K346T channels were transfected with Ha-tagged Ub and subjected to overnight MG132 treatment to induce inhibition of the proteosomal degradation. Kir2.1 was immunoprecipitated in treated and control cell lysates and ubiquitylation rate of the WT and K346T protein was revealed by immunoblotting (IB) versus Ub tag (Ha). Precipitation control was performed by IB using anti-Kir2.1 antibody (Supplementary Material, Fig. S4C and D). Densitometric analysis of the resulting bands showed a slightly lower ubiquitylation level for K346T compared with WT and proteasome inhibition by MG132 did not produce any accumulation of K346T protein in the cell (Supplementary Material, Fig. S4E and F), suggesting that the mutation could alter targeting of the protein to the proteasomal complex due to perturbation of physiological trafficking.

### The K346T mutation affects Kir2.1 channel compartmentalization in membrane lipid rafts

**Figure 5.**
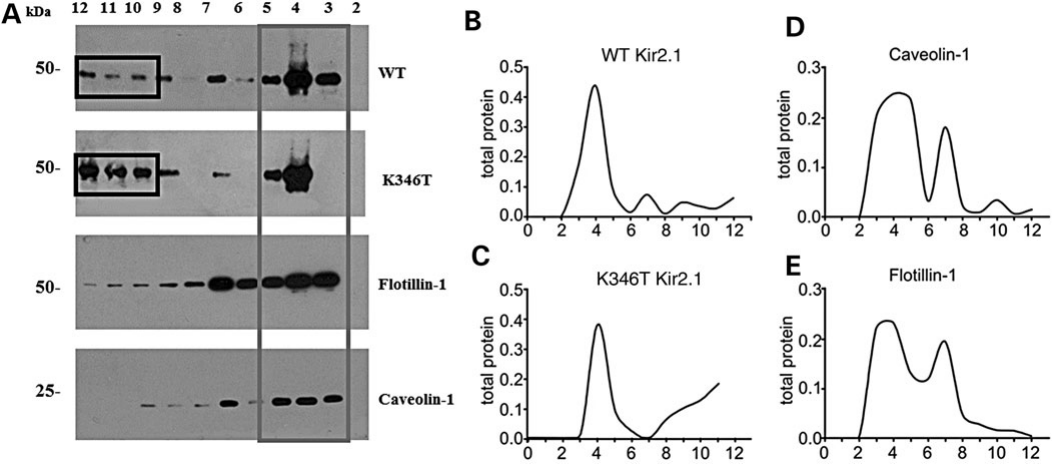
The K346T mutation affects the distribution of Kir2.1 channels in membrane lipid rafts. (**A**) WB analysis of cholesterol-rich (triton insoluble fractions: 3–5) and cholesterol-poor membrane fractions (triton soluble fractions: 10–12) of WT or K346T Kir2.1-expressing cells. WT channels are mainly distributed in triton insoluble fractions (gray box), whereas K346T is also abundantly localized in cholesterol-poor fractions (black boxes). Cav-1 and flotillin-1 identify the caveolar raft fractions. Molecular weight markers are on the left (kDa). (**B–E**) Normal distributions of total protein (indicated on top) in membrane fractions isolated by sucrose density gradient. The levels of protein in each fraction are normalized to the total protein amount recovered from all the fractions together.

Proteins degraded by the proteasome are mainly localized in ‘lipid rafts’, specific plasma membrane compartments enriched in cholesterol and internalized via ‘caveolae’, a subpopulation of rafts characterized by the presence of high levels of caveolin proteins forming flask-shaped membrane invaginations (26,27). Moreover, Ub binding to protein is known for triggering caveolin-mediated endocytosis (28). Previous studies have shown that Kir2.1 channels have a bimodal distribution between the raft and the non-raft membrane fractions (29,30). Kir2.1 channels partitioned into raft domains are in a more silent mode, whereas when they partition into non-raft domains, they enter into a more active mode (29,30). This is most likely caused by the different cholesterol content of each domain. Indeed, cholesterol has been shown to reduce Kir2.1 channel functionality by inducing a prolonged closed state of the channel (30). This notion prompted us to perform sequence analysis of Kir2.1 which showed that K346 (red residue in: YYKVDYSRFH**K**TYEV) resides in close proximity to both a cholesterol recognition/interaction amino acid consensus sequence (CRAC motif: V/L-X_1-5_-Y-X_1-5_-R/K—the underlined sequence above) and a caveolin-binding sequence [φXXXXφXXφ; φ: trp (W), Phe (F) or Tyr (Y)]. Based on this distinct body of evidence, we postulated that K346T could affect protein-lipid interactions and in turn alter the membrane partitioning of the channel. To test this hypothesis, we performed WB analysis on sucrose gradient-isolated cholesterol-rich (triton insoluble fraction) and cholesterol-poor membrane fractions (triton soluble fractions) of WT or K346T-expressing cells. Figure 5 shows the differential distribution of WT channels between low- and high-density membrane fractions, whereby they are more distributed in the triton insoluble fractions (Fig. 5A, gray box; Fig. 5B, fractions 3–5) as previously described (30). Conversely, the K346T mutation significantly enhanced the amount of protein localized in cholesterol-poor fractions (Fig. 5A, black boxes; Fig. 5C, fractions 10–12). The higher levels of cavolin 1 (Cav-1) and flotillin-1 (Fig. 5A, D and E) identify the caveolar lipid raft fractions enriched in cholesterol. These results demonstrated the presence of a larger population of K346T channels in cholesterol-poor fractions compared with WT and suggest that K346T-induced current density enhancement could also be due to reduced channel inhibition occurring because of the lower levels of cholesterol in these fractions. However, the molecular modeling and docking simulations of cholesterol revealed that K346T is located 10–14 Å away from the known and newly identified cholesterol-binding sites (Supplementary Material, Fig. S5).

### Kir2.1 interacts with Cav-1 and Cav-2 proteins

**Figure 6.**
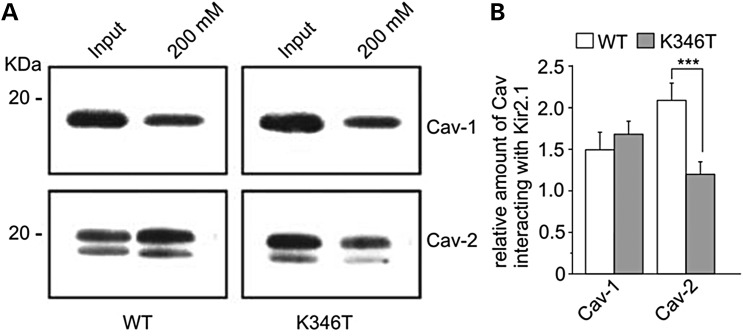
The K346T mutation reduces Kir2.1 channels interaction with Cav-2. (**A**) WB analysis of Kir2.1 channel's interactors after Histidine (His) co-purification of astrocytoma cells expressing WT or K346T channels. Input lanes represent protein extracts before His co-purification. WT and K346T channels co-purify similarly with Cav-1, whereas the K346T mutation reduces the association with Cav-2. One representative experiment out of three is shown. Molecular weight markers are indicated on the left (kDa). (**B**) Densitometric analysis (ratio) of protein bands corresponding to Cav-1 and Cav-2 normalized respect to WT (white bars) or K346T (gray bars) Kir2.1 protein levels. Data are expressed as mean ± SEM from three independent experiments (***P < 0.001).

The facts that (i) the K346T mutation also resides in the proximity of a putative caveolin-binding motif and (ii) caveolins influence cell surface expression, raft compartmentalization and trafficking of several type of K^+^ channels (31–33), prompted us to investigate whether Kir2.1 interacts with caveolin proteins that are expressed in cultured astrocytes (34), and the possible effects of K346T mutation. By performing the His-affinity co-purification assay described above, we found that Cav-1, the main structural component of caveolar rafts, similarly interacted with WT and K346T channels (Fig. 6A and B). In contrast, K346T mutation greatly reduced the association of Kir2.1 with Cav-2 (Fig. 6A and B), a protein directly involved in the regulation of cell signaling at raft levels (35). Cav-3, the muscle-specific caveolin isoform, could not be detected in U251 cells (M.S. Brignone, unpublished observation), confirming previous findings (34). Since Cav-1 and Cav-2 can modulate channel endocytosis leading to channel degradation or inactivation (31–33,36) and Cav-2 can also regulate membrane protein trafficking independently from Cav-1 (37), the results obtained here suggest that the differences in the associations with Cav-2 could influence K346T channels' membrane compartmentalization, stability and trafficking.

## DISCUSSION

In this study, we provide new *gain-of-function* mechanisms relevant to understand SQT3S pathogenesis, suggest the potential association of SQT3S with neurological disorders and uncover a multifunctional domain in Kir2.1 that controls pivotal properties of WT channels, such as surface expression, stability at the plasma membrane, partitioning to lipid rafts, ubiquitylation, protein degradation through proteasome and binding to Cav-2.

The K346T mutation resulted in larger homozygous and heterozygous K^+^ currents due to increased surface expression of the channel in oocytes, HEK293 and glial-like cells. The consistency of these results obtained with three very different expression systems would exclude cell type-bound biased conclusions and strengthen the notion that these effects occur in all mammalian cells that normally express this channel type namely myocytes, neurons and astrocytes. Notably, the mutation promotes the surface expression of the channels particularly at end-feet, filopodia-like structures and cell–cell contacts (Fig. 3). These structures are essential for astrocyte-mediated K^+^ siphoning through Kir2.1, Kir4.1 and Kir5.1 channels, all of which could be influenced by the K346T mutation.

It is now well established that the balance between trafficking pathways to and from cell membrane is an important determinant of steady-state cell surface density of membrane proteins. The time course of Kir2.1 surface expression and degradation suggest that the mutation hampers channel degradation and enhances surface expression also by means of protein stabilization at the plasma membrane. Such stability of the K346T channel does not appears to be due to altered interactions with syntrophin and α-dystrobrevin or Kir4.1, Kir5.1, Rac-1, Aquaporin-4 and connexin-43. However, the direct evidence provided here demonstrated that Kir2.1 channels are ubiquitylated and degraded through the Ub–proteasome pathway. The observation that a remarkably reduced amount of K346T protein accumulates upon proteasome inhibition with MG132, further supports the notion that the residue K346 influences protein's degradation/recycling. Furthermore, the fact that the K346T mutation did not completely abolish protein's ubiquitylation also suggests that other lysine residues may contribute to this process. High-throughput screening of rodent tissues has in fact indicated that ubiquitylation of Kir2.1 channels occurs at both K346 and K388 residues (38), the latter also being exposed intracellularly (see Supplementary Material, Fig. S6). Interestingly, ubiquitylation of Kir2.1 is tissue specific, in that it occurs in the brain but not in the heart and skeletal muscle of mice (38). This evidence suggests that K346T-induced deregulation of Kir2.1 ubiquitylation and proteasomal degradation may selectively alter brain, but not heart functions.

In general, membrane proteins localized in lipid rafts are mainly internalized via caveolae and degraded by the proteasome, whereas membrane proteins in non-raft areas of the membrane tend to be internalized via clathrin-coated pits and degraded in lysosomes. The mislocalization of K346T to non-raft areas of the membrane would thus reduce channel endocytosis via caveolar pathway and degradation by proteasome with the result of channel stabilization at plasma membrane. The implication of trafficking/endocytosis defects is further supported by the crucial observation that K346T channels exhibit a remarkably weaker interaction with Cav-2 compared with WT. This decreased interaction with Cav-2 and postulated decreased endocytic degradation or inactivation, would further account for the enhanced stability of K346T channels and mislocalization to non-raft regions of the plasma membrane.

Since the cholesterol content of a membrane negatively influences Kir2.1 current density due to conformational changes leading to prolonged closed states that cannot be detected by single-channel analysis (30,39), the demonstration that more K346T channels are distributed in cholesterol-poor fractions, compared with WT, can explain the larger current amplitudes recorded from oocytes, HEK293 and glial cells, all of which possess lipid rafts (40). Both the structural analysis of the residues known to affect the cholesterol sensitivity of several Kir channel types and the molecular docking simulations revealed novel-binding sites potentially involved in Kir2.1–cholesterol interaction (Supplementary Material, Fig. S5). This analysis also indicates that although the K346T is too far from these binding sites, it could still affect the intrinsic cholesterol sensitivity of the channels. Moreover, the location of the residue K346 is compatible with the involvement of this distinct intracellular domain in channel partitioning to lipid rafts, ubiquitylation, binding to Cav-2 and trafficking. Finally, our original finding that Cav-1 and Cav-2 associated with Kir2.1 represent an entirely new type of protein–protein interaction that may have important structural and functional implications.

### Potential implications for autism–epilepsy phenotype and SQT3 syndrome

Although it is formally possible that the *KCNJ2* mutation in *cis* with *KCNJ10* contributes separately to SQT3S or autism–epilepsy pathogenesis, each playing a clear distinctive role, this conclusion appears to be too simplistic. Kir2.1 channels are highly expressed in the brain, particularly in hippocampus, caudate, putamen, nucleus accumbens, habenula and amygdala (41), all areas implicated in cognition, mood disorders and ASD. As Kir2 channels, together with Kir4.1 and Kir5.1, contribute to regulate neuronal excitability, cell differentiation, synaptic plasticity and wiring, their dysfunction may impact these crucial neurophysiological processes and result in functional impairment of neural networks (further discussed in 11,12; 42–44). The clinical findings and mechanistic insights provided here, combined with recent studies showing the presence of neuropsychiatric disorders in individuals with mutations in *KCNJ2* (2,4–6), indicate a possible role of the Kir2.1 channels in the pathogenesis of autism–epilepsy. Given that most ASD behave as a complex multigenic disorder, Kir2.1 dysfunction in limbic neurons and astrocytes may enhance susceptibility to the disease when other contributing alleles (including *KCNJ10*, as in our probands) are co-inherited.

In hippocampus, the amplitude of Kir2.1 currents is small in young dentate granule neurons (DGCs) and increases ∼3× in mature DGCs to optimize their excitability and, therefore, Kir2.1 plays an important role in DGCs firing properties during development (45). With regard to seizures, it has been proposed that Kir2.1 upregulation in DGCs would counterbalance the hyperexcitability observed in temporal lobe epilepsy and thus function as an anti-convulsant (46). On the other hand, upregulation of Kir2.1 channels has been observed in hippocampal astrocytes following kainic acid-induced seizures (8). Thus, whether Kir2.1 channels function as anti-convulsant or proconvulsant is unclear. Intriguingly, in both twins seizures had a short course and EEGs normalized by the age of 3 years (11).

The ECG recordings and the molecular diagnosis provided here (Fig. 1) demonstrated that both monozygotic twins suffered from SQT3S, presumably resulting from larger I_K1_ currents. These are thought to be predominantly carried, in the heart, by Kir2.1 channels which contribute to fine-tune the resting membrane potential and the final phase of action potential repolarization. The electrophysiological changes of I_K1_ properties caused by the K346T mutation are very similar to those of the other *KCNJ2* mutation found in SQT3S (i.e. D172N; 8) and atrial fibrillation (47), indicating that K346T likely contributes to arrhythmia generation by affecting the excitability of myocytes. In particular, a reciprocal modulation of Kir2.1 and N_av_1.5 channels seems to be relevant to self-sustained cardiac rhythm disturbances (48). Whether *gain-of-function* mutations in Kir2.1 enhance the availability of N_av_1.5 in neurons, and if this mechanism might contribute to lowering the threshold for seizures\ASD remains an intriguing hypothesis. Notably, the association of cardiac arrhythmias with autism, as seen in our twins, is not entirely unexpected. As a matter of fact, the phenotype of Timothy syndrome (OMIM 601005) involves multiple organs, including heart and brain, and is characterized by long QTc intervals (400–700 ms), lethal cardiac arrhythmia, seizures and ASD in over 80% of the patients (49–51). Thus, the Kir2.1 functional defects reported here emerge as potentially crucial for astrocytes dysfunction and suggest careful assessments for comorbid neuropsychiatric disturbances in patients with inherited arrhythmogenic diseases caused by Kir2.1 channel dysfunction. Finally, this study also raises the question as to whether (regardless of the distinct *gain-of-function* mutation causing SQT3S), hypocholesterolemia would contribute to trigger SQT3 arrhythmic episodes by further increasing Kir2.1 availability, or if, vice versa, borderline hypercholesterolemia would reduce the severity of symptoms. These assumptions, though logical in the setting of our experimental approach, deserve further investigations in more appropriate clinical settings given their potential impact on disease management and therapeutics.

## MATERIALS AND METHODS

### Genetic analyses

Total genomic DNA was purified from peripheral blood and the coding exons and exon–intron boundaries of *KCNJ2* (NM_000891.2, NG_008798.1) were amplified by polymerase chain reaction (PCR) using specific oligonucleotides primer (PCR conditions upon request). The PCR products were bidirectionally sequenced using the BigDye v3.1 chemistry (Applied Biosystems Foster City, CA, USA). Multiple alignments with Kir2.1 orthologs were performed using ClustalW (www.ebi.ac.uk/clustalw/) to evaluate the degree of conservation of missense variants and Polyphen modelling analysis (http://genetics.bwh.harvard.edu/pph/) was used to predict their effects *in silico*. Healthy, ethnically matched control chromosomes were screened by direct sequencing. Legal representatives of patients signed informed consent prior to enrolment. The local Institutional Review Board approved this study.

### Expression of Kir2.1 channels in Xenopus oocytes

The human Kir2.1 cDNA was introduced into in the pBF oocyte expression vector and the K346T mutation was generated by site-directed mutagenesis. Capped mRNAs were synthesized, *in vitro*, as previously described (52–54). *Xenopus laevis* were deeply anesthetized with an aerated solution containing 3-aminobenzoic acid ethyl ester methansulfonate salt (5 mm) and sodium bicarbonate (60 mm), pH = 7.3. To further reduce their suffering, *X. laevis* underwent no more than two surgeries, separated by at least 3 weeks. Stages IV–V Xenopus oocytes were isolated, injected with 50 nl mRNAs and stored at 16°C in fresh ND96 medium containing (mm): NaCl 96, KCl 2, MgCl_2_ 1, CaCl_2_ 1.8, HEPES 5, gentamicin 50 μg/ml (Sigma, Italy). Procedures involving *X. laevis* and their care were in accordance with the regulations of the Italian Animal Welfare Act and were approved by the local Authority Veterinary Service, in agreement with the NIH Guide for the Care and Use of Laboratory Animals. The minimal number of animals was used. mRNA concentrations were quantified by electrophoresis and ethidium bromide staining and by spectrophotometric analysis. Equal amount of either WT or mutant mRNAs were then microinjected into Xenopus oocytes according to standard protocols.

### Expression of Kir2.1 channels in HEK293 cells

The K346T mutant was expressed in HEK293 cells by using the same approach described previously (8). The *KCNJ2* mutation was engineered into WT cDNA cloned in pcDNA3.1 (Invitrogen, Life Technologies, Carlsbad, CA, USA) and confirmed by sequence analysis. HEK 293 cells were transfected with 1.6 μg plasmid DNA of Kir2.1 WT or K346T mutant using Effectene (Qiagen, Hilden Germany), as directed by the manufacturer. To mimic the heterozygous substrate, 0.8 μg of each plasmid was transfected. In addition, 0.8 μg of GFP cDNA was co-transfected to serve as a reporter gene.

### Generation of U251 astrocytoma cell lines overexpressing His-tagged Kir2.1 WT or carrying p.K236T mutation and cell line treatments

Astrocytoma U251MG cell line was obtained from the American Type Culture Collection (Rockville, MD, USA) and grown in Dulbecco's modified Eagle's medium high glucose (Euroclone, Ltd., UK) supplemented with 10% FBS (Gibco BRL, Paisley, UK), 1% penicillin/streptomycin (Sigma Ltd, Irvine, UK) at 37°C in a 5% CO_2_/95% air atmosphere. To generate U251 astrocytoma cell lines overexpressing His-tagged Kir2.1 channels, the WT and K346T cDNAs were cloned into pcDNAV4-HIS, as previously reported (24), by RT-PCR amplification (PCR conditions upon request). After sequencing analysis Kir2.1, cDNAs were subcloned into a retroviral bicistronic vector (pQCXIN, Takara Bio Europe Clontech, France) and transfected into a packaging cell line (GP2, HEK293) to generate replication incompetent retroviral particles. Viral suspensions were then used to infect U251MG astrocytoma cell lines as described (55). Cells infected with virus carrying the empty vector as control (mock-infected U251) or overexpressing recombinant proteins (WT or K346T) were obtained by growing in G418 (Gentamicin, Euroclone) containing selective medium at a concentration of 600 μg/ml. For cell treatments, astrocytoma cell lines were plated in 100-mm diameter dishes and treated for different time lengths (3 h, 6 h, overnight) with cycloheximide (100 μg/ml, Sigma). After stimulation, cells were collected and solubilized as described below. Proteins were analyzed by SDS–PAGE and WB.

### Electrophysiology

TEVC recordings were performed from oocytes at room temperature (22°C) and, 1–8 days after injection, by using a GeneClamp 500 amplifier (Axon Instruments, Foster City, CA, USA) interfaced to a PC computer with an ITC-16 interface (Instrutech Corporation, Longmont, CO, USA). Microelectrodes were filled with KCl 3 m. To avoid clamping artifacts, the current-passing electrode was placed near the center of the cell, and low resistance microelectrodes (∼0.1 MΩ) were used for the short-duration recordings (56). Standard bath solution contained 90 mm KCl, 3 mm MgCl_2_, 10 mm HEPES (pH 7.4). Recordings were filtered at 2 kHz and acquired at 5 kHz with Pulse software and analyzed with either PulseFit (HEKA, Germany) or IGOR (WaveMetrics, Lake Oswego, OR, USA). Currents were evoked by voltage commands from a holding potential of −10 mV, delivered in −10 mV increments from +50 to −120 mV, unless otherwise stated.

Patch-clamp recordings of Xenopus oocytes were performed at 22°C using an Axopatch 200B amplifier (Axon Instruments) as previously described (54). Oocytes were bathed in a solution containing 120 mm KCl, 1 mm CaCl_2_, 11 mm EGTA, 10 mm HEPES, 0.1 mm dithiothreitol (pH 7.2) and had resting membrane potentials (Vm) of ∼0 mV in this ionic conditions. Recording electrodes were pulled from borosilicate glass, dipped in sticky wax (Kerr, Emoryville, CA, USA) prior to polishing and had resistances of 3–8 MΩ. The pipette solution, used for single-channel recordings, contained 120 mm KCl, 10 mm HEPES, 200 μm CaCl_2_ (pH 7.2). The use of high potassium concentrations in the pipette was necessary to clearly resolve inward unitary currents. Patch-clamp recordings were performed in the cell-attached configuration by stepping to various test potentials and assuming that the Vm of the cell was ∼0 mV. Junction potentials between bath and pipette solutions were properly nullified. Current traces at each holding potential were filtered at 1 kHz with a 4-pole low-pass Bessel filter and acquired at 5–10 kHz with a Pulse+PulseFit program (HEKA Elektronik GmbH, Germany). Channel activity was analyzed with a TAC-TAC fit program (Bruxton Co., Seattle, WA, USA) using the 50% threshold technique to determine the event amplitude. Channel openings were visually inspected before being accepted (event-by-event mode).

Patch-clamp recordings of HEK293 or U251MG cells were performed by using an Axopatch 700B or 200B Amplifiers (Axon Instruments), at room temperature. The extracellular recording solution contained (in mmol/l) NaCl 135, KCl 4.8, CaCl_2_ 1.8, MgCl_2_ 1, Glucose 10 and HEPES 5; pH was adjusted to 7.4 with NaOH. The micropipette solution contained (in mmol/l) KAsp 130, KCl 15, MgCl_2_ 1, K2-ATP 2 and HEPES 5; pH was adjusted to 7.4 with KOH. To show Kir2.1 specificity, 1 mmol/l BaCl_2_ was added to the bath solution to block the inward rectifying current. IK1 data were plotted as barium-sensitive currents. Data were adjusted for the liquid junction potential (15 mV) and presented as mean ± SEM. Two-tailed Student's *t*-test was used to compare means; *P* < 0.05 was considered statistically significant.

### Immunofluorescence and confocal microscopy analyses

Cells were grown subconfluent on polylysine-coated coverslips, fixed for 10 min with 4% paraformaldehyde and washed with PBS. After 1 h of incubation with blocking solution (5% BSA in PBS), cells were incubated for 1 h at room temperature with affinity purified anti-Kir2.1 polyclonal antibody (pAb, 1:50, Alomone, Jerusalem, Israel) diluted in PBS, 0.025% Triton X-100. As secondary Ab, we used TRITC-conjugated goat anti-rabbit IgG H+L (Jackson Immunoresearch Laboratories, West Grove, PA, USA). To stain actin filaments, an NBD phallacidin high-affinity F-actin probe (1:30, Invitrogen, Life Technologies, Monza, Italy) was used in combination with the primary Abs. Coverslips were washed, sealed in Vectashield medium (Vector Lab, Burlingame, CA, USA) and analyzed with a laser scanning confocal microscope (LSM 5 Pascal, Carl Zeiss, Jena, Germany).

### Co-purification of histidine-tagged proteins

Lysates obtained from two 175 cm^2^ flasks of confluent astrocytoma cell lines stably overexpressing His-tagged WT and mutated Kir2.1 and mock-infected control (U251) cells were incubated overnight at 4°C with 200 µl (50%, v/v, suspension) of Ni-NTA Agarose (Qiagen, Hilden, Germany). After extensive washings (10 bed volumes of 10–25–50 mm Imidazole, 0.5% Triton X-100, 150 mm NaCl, 20 mm Tris–HCl, pH 7.4), protein elution was carried out using 200 mm imidazole (24). Eluted proteins were precipitated with acetone (1:4, v/v) and analyzed by SDS–PAGE and WB.

### Detergent-resistant microdomain (DMR/lipid rafts) preparation by sucrose gradients

DRMs from cultured astrocytoma cell lines overexpressing WT and mutated Kir2.1 were prepared as previously described (57). Briefly, human astrocytoma cell lines were grown to confluence in 100-mm dishes, harvested and lysed on ice with 0.75 ml of Mes-buffered saline (25 mm MES, pH 6.5, 0.15 m NaCl) containing 1% (v/v) Triton X-100 and protease inhibitors. Cell lysate was homogenized with 10 strokes of a Dounce homogenizer, adjusted to 40% sucrose and placed at the bottom of an ultracentrifuge tube. A 5–30% linear sucrose gradient was placed above the homogenate and the mixture was centrifuged at 60 000*g* for 16 h at 4°C in a SW 61 rotor (Beckman Instruments). Twelve 0.375-ml fractions were harvested from the top of the gradient. The DMR fractions are visible as a light-scattering band migrating at ∼20% sucrose (fractions 3, 4 and 5). Samples were precipitated over night with acetone (1:4, v/v) and proteins analyzed by SDS–PAGE and WB.

### Protein extract preparation and WB

Astrocytoma cell lines were lysed and analyzed by WB as previously described (24,57). For protein detection, the following Abs were used: anti-Kir2.1 pAb (1:250, Alomone, Israel), anti-Kir4.1 pAb (1:400, Alomone), anti-actin mAb (1:2000, Santa Cruz Biotecnology, Inc., Santa Cruz, CA, USA), anti-Cav-1 pAb (1:1000, Santa Cruz Biotecnology), anti-Cav-2 pAb (1:3000, Abcam, Cambridge, UK), anti-Kir5.1 pAb (1:500, Abcam), anti-flotillin mAb (1:1000, BD Transduction Laboratories), anti-connexin-43 mAb (1:250, BD Transduction Laboratories), anti-syntrophin mAb (1:200, MA-1-745, Affinity BioReagents, CO, USA), anti-dystrobrevin mAb (1:750, BD Transduction Laboratories), anti-RAC1 mAb (1:3500, BD Transduction Laboratories) and anti-Ub (P4D1) mAb (1:200, Santa Cruz Biotecnology) in PBS+3% BSA and then incubated with horseradish peroxidase-conjugated anti-mouse or anti-rabbit Ab (1:10 000; Thermo Scientific, Missouri, MO, USA), for 1 h at RT. Immunoreactive bands were visualized using an enhanced chemiluminescence reagent (Pierce, Thermo Fisher Scientific, Rockford, IL, USA), according to the manufacturer's instructions and exposed on X-ray films.

### *In vivo* ubiquitylation assays

U251 cells were transfected with a CMV driven HA-Ub plasmid (gift of Prof D. Bohmann) using Lipofectamine LTX and Plus reagent (Life Technologies) according to the manufacturer's instructions. Twenty-four hours posttransfection cells treated with 10 μm MG132 (Sigma–Aldrich) for 16 h were trypsinized, neutralized with complete medium and washed with PBS. For immunoprecipitation of ubiquitinated WT and K346T mutant, cells were lysed in protease inhibitors containing RIPA buffer. Lysates were clarified and 1 mg of protein were precipitated with 1:1000 mouse mAb to Xpress (Invitrogen, Life Technologies) or 1:500 Histidine tag mAb (Abcam) and 1:250 rabbit pAb to Kir 2.1 (Alomone). Immunocomplexes recovered with protein G-Sepharose (GE Healthcare, Milan, Italy) were washed five times with Net Gel Buffer and boiled in 25 μl of Laemmli buffer 2× for 5 min. Resulting immunocomplexes were resolved on 8%–12% discontinous gradient SDS–PAGE and transferred to nitrocellulose membrane (Bio-Rad, Milan, Italy). Membranes were probed with mAb to HA (Cell Signaling) and pAb to Kir2.1 (Alomone) and detected using HRP-conjugated secondary antibodies (Bio-Rad) and ECL WB reagent for chemiluminescence (Thermo Scientific).

Densitometric analyses of WB experiments were performed using NIH ImageJ software. Ub bound was normalized to the total immunoprecipitated Kir 2.1 amount.

## STATISTICAL EVALUATION

Data are presented as means ± SEM. The significance of differences between groups was investigated by ANOVA and Student's *t*-test, and the following levels of significance noted *P < 0.05; **P < 0.02; ***P < 0.001.

### Homology modeling and cholesterol docking

The 3D structure of Kir2.1 was built by comparative modeling using the software Modeller^®^ (58). The X-ray structure of the Kir3.1-prokaryotic Kir channel chimera (PDB id.: 3JYC) was used as a template. Sequence alignment of the target sequence versus the template was generated using ClustalX, and further refined using Muscle (59). The percentage of identity on the aligned sequence was 36.7%, whereas the similarity was 66.3%; only residues 25–349 of the Kir4.1 primary structure and residues 31–347 of the Kir5.1 sequence could be aligned with the corresponding stretches in the X-ray template. Twenty homology models were generated and scored against the minimum number of constraint violations. Among them, the five lowest energy models were selected and analyzed using Procheck (http://www.ebi.ac.uk/thornton-srv/software/PROCHECK/; 60). The final model was selected according to the highest percentage of residues in the allowed region of the Ramachandran plot (>90%). The model was then immersed in a pre-equilibrated patch of POPC lipids bilayer and all overlapping lipid molecules (within 3 Å from any protein atoms) were removed. Finally, the mutant protein in Kir2.1 was generated by substituting the side chain of lysine-346 with threonine using VMD software (www.ks.uiuc.edu/Research/vmd/; 61) and the resulting structure was further minimized to reduce steric hindrance with neighboring atoms. Preparation of the data, including addition of hydrogens to the ligand and the receptor, determination of the rotatable bonds, partial charge distribution via the Gasteiger method (62), definition of the region of Kir2.1 in which to execute the docking and the grid calculation for the docking algorithms, was done with the AutoDockTools 1.5.4 program (63). The channel molecule was firstly energy minimized using steepest descent algorithm. Docking of cholesterol was done using the Lamarckian Genetic Algorithm protocol implemented in Autodock 4.2 (64). A 60 × 60 × 60 Å^3^ box was built around L222 to find potential cholesterol-binding sites within this box. A total of 150 runs were carried out to obtain 50 different configurations of cholesterol bound to the Kir2.1-binding site. To obtain a large number of different conformations of bound cholesterol, only runs that resulted in an RMS difference >2 Å were considered. During the docking procedure, all rotatable bonds in the cholesterol molecule were allowed to rotate. The final selected conformations of docked cholesterol were chosen based on a cluster analysis of all the 50 conformations using a 0.5 Å cutoff.

## SUPPLEMENTARY MATERIAL

Supplementary Material is available at *HMG* online.

## FUNDING

This work was supported by Telethon grant (GGP11188), MIUR-PRIN (20108WT59Y_004), COMPAGNIA di San Paolo (Turin) ‘*Programma Neuroscienze*’, Ministero della Salute (GR-2009-1580433) and Fondazione Cassa di Risparmio di Perugia, Telethon grants GGP11141 and GGP06007 (to S.G.P.), Fondation Leducq Award 08CVD01 (to S.G.P.), by Fondazione Veronesi Award on inherited arrhythmogenic diseases (to S.G.P.).

## Supplementary Material

Supplementary Data
