# Supplementary material for: Genetically induced dysfunctions of Kir2.1 channels: implications for short QT3 syndrome and autism–epilepsy phenotype
Source: Hum Mol Genet. 2014 May 2;23(18):4875–86. doi: 10.1093/hmg/ddu201 (PMC4140467; doi:10.1093/hmg/ddu201)
Supplement: Supplementary Data [file supp_23_18_4875__index.html]

Genetically induced dysfunctions of Kir2.1 channels: implications for short QT3 syndrome and autism–epilepsy phenotype — Genetically induced dysfunctions of Kir2.1 channels: implications for short QT3 syndrome and autism–epilepsy phenotype — Genetically induced dysfunctions of Kir2.1 channels: implications for short QT3 syndrome and autism–epilepsy phenotype — Supplementary Data 

# Genetically induced dysfunctions of Kir2.1 channels: implications for short QT3 syndrome and autism–epilepsy phenotype

## Supplementary Data

Supplementary Data

**Files in this Data Supplement:**

- Supplementary Data - Pdf file
