# Supplementary material for: Genetically induced dysfunctions of Kir2.1 channels: implications for short QT3 syndrome and autism–epilepsy phenotype
Source: Hum Mol Genet. 2014 May 2;23(18):4875–86. doi: 10.1093/hmg/ddu201 (PMC4140467; doi:10.1093/hmg/ddu201)
Supplement: Supplementary Data [file supp_ddu201_ddu201supp.pdf]

SUPPLEMENTAL DATA

Clinical findings

The two 9-year-old identical twins born from unrelated parents (Fig. 1A) experienced, since 5 months of age, impaired social interaction, sleep difficulties and hypotonia. At age 7 months, within 24 hours, both infants manifested epileptic spasms that ceased under ACTH at 10 months. One of the twins also displayed focal seizures from 10 to 12 months of age, which remitted under valproate-topiramate therapy. Neurological examination at the age of 10 months showed axial hypotonia, nystagmus, and absence of speech. On subsequent follow-up, the severe impairment of social interaction and communication, associated with stereotypes and repetitive behaviours, were consistent with DSM-IV-TR criteria for ASD. The latest examination at age 8 years was also significant for symptoms of anxiety, depression, obsessive-compulsive disorder and mild intellectual disability. On routine clinical evaluation, both children showed an electrocardiogram (ECG) with a markedly short repolarization time and conspicuously narrow and peaked T waves (QTc interval, 331 ms) (Fig. 1B). Unfortunately, the parents refused any further investigation besides the standard resting ECG. The 49-year-old mother had a history of presyncopal episodes and palpitations, as well motor clumsiness, obsessive-compulsive symptoms, mood swings with impulsivity, and motor and vocal tics fitting the diagnosis of Tourette Syndrome.

Electrophysiological characterization of K346T channels expressed in HEK293 cells

Currents were elicited by 400-ms depolarizing voltage steps from -100 to +40 mV and from a holding potential of -70 mV. At potentials between −65 and −55 mV, both homotetrameric (K364T) and heterotetrameric (WT/K364T) channels presented larger outward currents compared to WT, when recorded at physiological extracellular and intracellular K^+ concentrations (*see experimental procedures*). The effect of the K364T mutation is similar to

that of D172N even if within narrower windows (−65 and −55 mV for K364T and −75 and −55 mV for D172N), which may suggest a milder increase of repolarizing current. Current densities for the WT (n=12), K346T (n=14) and WT/K346T (n=16) were 16.3±2.1pA/pF, 33.5±2.4 pA/pF, 24.9±2.5 pA/pF at a test potential of -65 mV, respectively. There were no significant changes in reversal potentials (WT=−86.2±1.9 mV; K346T=−87.1±1.4 mV; WT/K346T=−86.7±1.3 mV) and rectification properties.

pH sensitivity of WT or K346T channels

Extensive studies have shown that lysine residues function as pH modulators of Kir channel activity (Pessia *et al.,* 2001; Hibino *et al.,* 2010). We thus determined the pH sensitivity of WT channels by using a well established K-acetate buffering system which has been shown to modify the intracellular pH (pH_i) of oocytes (Choe et al., 1997; Tucker et al., 2000; Pessia *et al.,* 2001; Casamassima et al., 2003). The perfusion of oocytes with a K-acetate buffer, which has been shown to reduce the pH_i to 6.4 (14), inhibited 13.9±1.5% and 10.7±0.7% the current amplitudes of WT and K346T, respectively (n= 4; $p>0.05$). Extracellular acidification was unable to modify the whole-cell amplitude of WT or K346T currents (*not shown; Ilenio Servettini*). Collectively the data ruled out the likelihood that the p.K346T mutation could exert pathogenic effects by altering the pH_i sensitivity of the channel, leaving the increased surface expression of K346T channels as the most likely pathogenic alternative.

The K346T mutation affects ubiquitin binding and proteasome-dependent degradation of Kir2.1 protein

Since Ubiquitin (Ub) plays an essential role in the degradation of membrane proteins and generally Ub binding occurs between a lysine of the target protein and the C-terminal glycine of ubiquitin, the involvement of a lysine residue in Kir2.1 stability prompted us to verify whether

ubiquitylation could play a role in this process. To evaluate the background ubiquitylation levels of recombinant WT and K346T proteins, we performed WB analysis with anti-polyubiquitin and anti-Kir2.1 antibodies on WT and K346T protein eluates derived from His pull-down assay on astrocytoma cells (Fig. S5A). These experiments revealed that Kir2.1 is ubiquitylated and that that the ubiquitylation levels for K346T channels were lower than the WT (Fig. S5A,B). Similar data were obtained by using an *in vitro* ubiquitylation assay. Cells expressing WT or K346T channels were transfected with Ha *tagged* Ub and subjected to an overnight MG132 treatment to induce inhibition of the proteosomal degradation. Kir2.1 was immunoprecipitated with anti-HA Ab in treated and control cells lysates and, after SDS/PAGE separation, ubiquitylation rate of the WT and K346T protein was revealed by immunoblotting (IB) *vs* ubiquitin tag (Ha) (Fig. S5C-E). Densitometric analysis of the resulting bands showed a slightly lower ubiquitylation level for K346T compared to WT (Fig. S5F) and indicated that proteasome inhibition by MG132 did not produce any accumulation of K346T protein in the cell (Fig. S5F).

Molecular modelling and docking simulations of cholesterol.

To verify whether K346 could affect directly both the binding and sensitivity of the channel to cholesterol we first generated a 3D-homology model of a Kir2.1 channel, using available crystal structure data as a template (Tao et al., 2009), localised the K346 and performed in silico mutagenesis. This analysis revealed that the side-chains of either K346 or T346 are exposed to the cytoplasm (Fig. S6). The relevant residues that have been shown to affect the cholesterol sensitivity of several Kir channel types (Rosenhouse-Dantsker et al., 2011) were also located and depicted in figure S6, which reveals that they form a distinct cytosolic belt. Furthermore, the molecular docking of cholesterol to the channel was performed (*SI methods*). This analysis indicated that the pK346T resides more than 20Å away from the residues that affect either cholesterol-induced inhibition of channel activity or cholesterol binding (Fig. S6).

SUPPLEMENTAL FIGURES

Supplementary Figure 1

Single-channel current recordings of WT and K346T channels.

Representative single-channel current recordings of WT (***A***) and K346T (***B***) channels obtained at -80 mV in a cell-attached configuration of the patch-clamp. The bottom traces are shown on an expanded time scale. Channel openings are downward deflections and arrows denote closed (C) and open (O) levels (*horizontal dashed lines indicate 0 current level*). Current traces were filtered at 0.5 kHz. Averaged sweeps with no openings were used to subtract leak and capacity currents. (***C***) Averaged single-channel current amplitudes were plotted as a function of voltage to calculate the slope conductance. This analysis revealed no significant differences between WT (42.0 pS; *black circles*) and K346T (38.8 pS; *red circles*) single-channel conductance (mean±SEM; n=6; p□0.05).

Supplementary Figure 2

Electrophysiological recordings from U251 cells expressing either WT or K346T channels.

(***A***) Sample current families recorded from astrocytoma U251MG cells expressing WT (*top*) or K346T (*bottom*) channels. Voltage-command pulses evoked typical Kir2.1 currents with strong rectification. The steps were delivered from a holding potential of -70 mV in -10 mV increments from -30 to -140 mV (*the pulse protocol is shown as inset in* ***A***). (***B***) Representative steady-state current density (pA/pF) as a function of voltage for WT (*black circles*) and K346T channels (*red circles*).

Supplementary Figure 3

Kir2.1 channel's interactors.

WB analysis of Kir2.1 channel interactors identified by Histidine (His) co-purification of astrocytoma cells expressing His-tagged WT or K346T channels. Eluates derived from His pull-down, performed with astrocytoma cells infected with the empty vector, are used as a control for non specific binding to NiNTA-resin (U251). Input lanes represent the starting protein extracts before His pull-down. Interactors have been eluted from NiNTA-resin using imidazole (200 mM). WT and K346T channels co-purify similarly with syntrophin (*synt*), dystrobrevin (DB), while no interaction is observed with the Rho GTPase Rac-1. Unexpectedly, among Kir2.1 interactors we identified Kir4.1 and Kir5.1 subunits that were found equally in both WT and K346T expressing cell extracts. One representative experiment out of four is shown. Molecular weight markers are indicated on the left (kDa).

Supplementary Figure 4

The K346T mutation affects proteasome-dependent degradation of Kir2.1 protein.

(***A***) His-tagged WT and mutated Kir2.1 derived from His pull-down assay of astrocytoma cells after elution from NiNTA-resin using imidazole (200 mM, as described above), were resolved by SDS/PAGE, and ubiquitylation levels assessed by WB analysis. A reduction in ubiquitylation levels was detected for K346T compared to WT channels when the eluted proteins were loaded and blotted with anti-polyUb mAb followed by anti-Kir2.1 pAb. Input lanes represent the starting protein extracts before His pull-down. (***B***) Histogram reporting the densitometric analysis of the ubiquitylated Kir2.1 bands in the eluates normalized versus the total amount of Kir2.1 protein of either WT (*grey bar*) or K346T (*red bar*). Data are expressed as mean±SEM from 4 independent experiments. (***C***) WT and K346T espression in Ha-ubiquitin transfected astrocytoma cells. Following MG132 treatment, WB analysis showed higher levels of mutant

protein respect to WT with accumulation after proteasomal block. (***D***) Transfection control for Ha-tagged ubiquitin was performed before MG132 treatment, and α-tubulin was used as loading control. (***E***) *In vitro* ubiquitylation assay in astrocytoma cells. Immunoprecipitation analysis were performed with cells transfected with Ha-tagged ubiquitin and treated with MG132 to inhibit proteasome targeted degradation. Immunoprecipitation of protein extracts with anti-His antibody was used to recover Kir2.1 immunocomplexes, and resulting material was examined by WB and probed using anti-Ha antibody to detect the ubiquitinated proteins. Samples were probed for protein IP efficiency using anti-Kir2.1 antibody. (***F***) Densitometric analysis of WB from *in vitro* ubiquitylation. Ubiquitylation smears were normalized versus the total immunoprecipitated amount of either WT or K346T protein. Data are expressed as mean±SEM from three indipendent experiments (***p <0.001).

Supplementary Figure 5

Homology modelling and cholesterol docking to Kir2.1 channels.

Stereoview of the homology model for Kir2.1 channels (*left*). Horizontal dashed lines delimit the membrane spanning region (trans-membrane (TM), extra- (EC) and intra-cytoplasmic (IC) regions). (*Right*) Close-up view of the intra-cytoplasmic region of Kir2.1 showing two adjacent subunits. The K346 residue (*green*) and the K346T mutation (*red*), performed *in silico*, are shown in two adjacent subunits. The location of the "*cholesterol binding pocket*" is shown with a representative cholesterol moiety (*red, orange and yellow sticks*) for the top four clusters (*see methods*). The cytosolic belt of residues that affect the cholesterol sensitivity of Kir channels are shown as grey spheres (Rosenhouse-Dantsker et al., 2011).

Supplementary Figure 6

Localization of the residue K338 in Kir2.1 channels.

(*Right*) Stereoview of the homology model for Kir2.1 channels. (*Left*) Close-up view of the intra-cytoplasmic region of Kir2.1 showing the residue K388 (*yellow*) and K346T (*red*).

SUPPLEMENTAL REFERENCES

Choe, H., Zhou, H., Palmer, G., Sackin, H. (1997) A conserved cytoplasmic region of ROMK modulates pH sensitivity, conductance, and gating. *Am. J. Physiol.* **273**(4 Pt 2),F516-529.

Tucker, J.S., Imbrici, P., Salvatore, L., D'Adamo, M.C., Pessia, M (2000) pH-Dependence of the Inwardly Rectifying Potassium Channel Kir5.1 and Localisation in Renal Tubular Epithelia. *J. Biol. Chem* .**275,**16404-16407.

Casamassima, M., D'Adamo, M.C., Pessia, M., Tucker, S.J. (2003) Identification of heteromeric interaction which influences the rectification, gating and pH-sensitivity of Kir4.1/Kir5.1 potassium channels. *J. Biol. Chem.* **278,**43533-43540.

Tao, X., Avalos, J.L., Chen, J., MacKinnon, R. (2009). Crystal structure of the eukaryotic strong inward-rectifier K+ channel Kir2.2 at 3.1 A resolution. *Science***.** **2009** 326(5960),1668-74.

Rosenhouse-Dantsker, A., Logothetis, D.E., Levitan, I. (2011) Cholesterol sensitivity of KIR2.1 is controlled by a belt of residues around the cytosolic pore. *Biophys. J.* **100(**2),381-389.

Pessia, M., Imbrici, P., D'Adamo, M.C., Salvatore, L., Tucker, S.J.(2001) Differential pH-sensitivity of Kir4.1 and Kir4.2 and Modulation by Heteropolymerisation with Kir5.1. *J. Physiol.* **532** (Pt 2),359-367.

Hibino, H., Inanobe, A., Furutani, K., Murakami, S., Findlay, I., Kurachi, Y. (2010) Inwardly rectifying potassium channels, their structure, function, and physiological roles. *Physiol. Rev.* **90**(1),291-366.

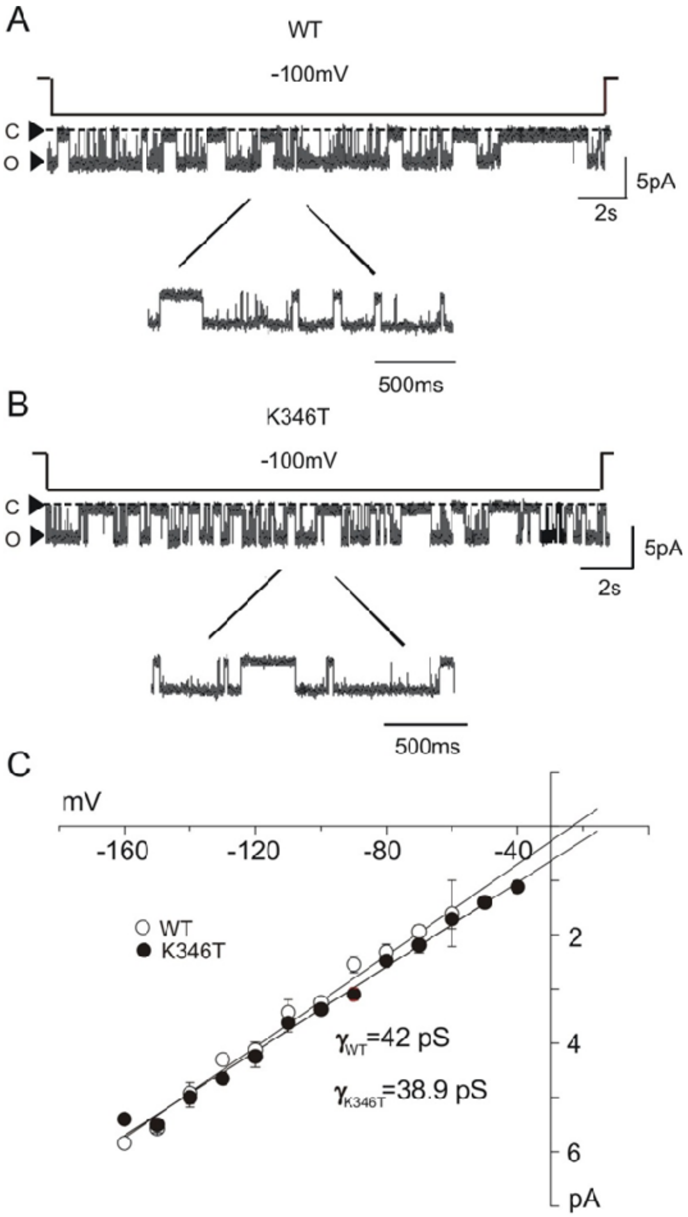

Supplementary figure S1

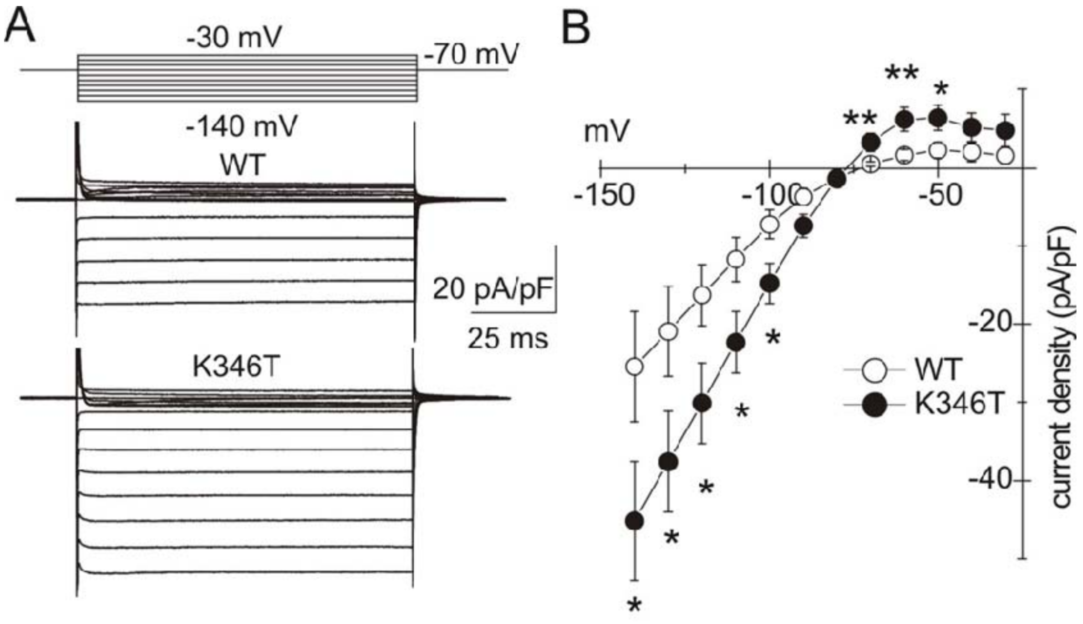

Supplementary figure S2

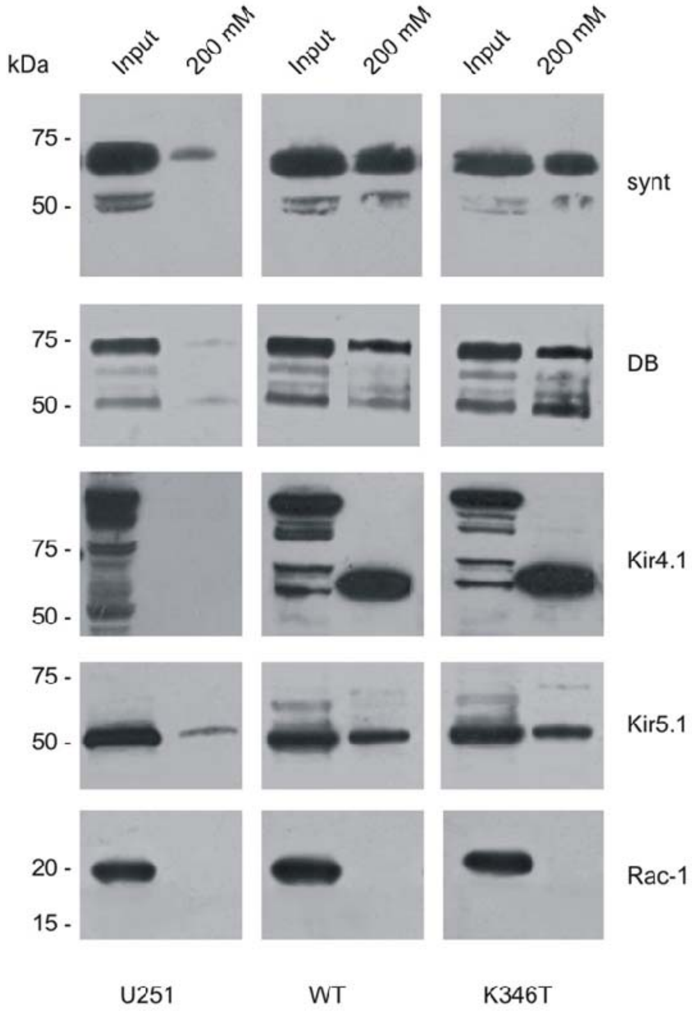

Supplementary figure S3

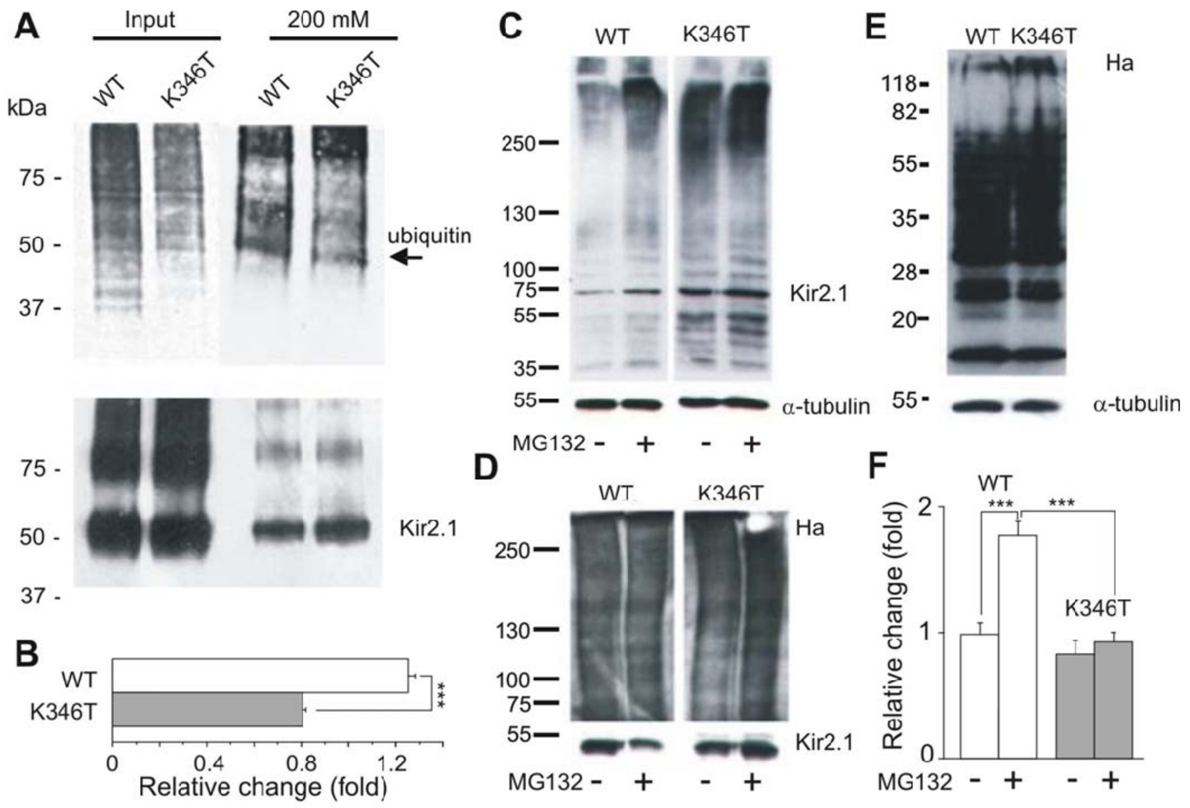

Supplementary figure S4

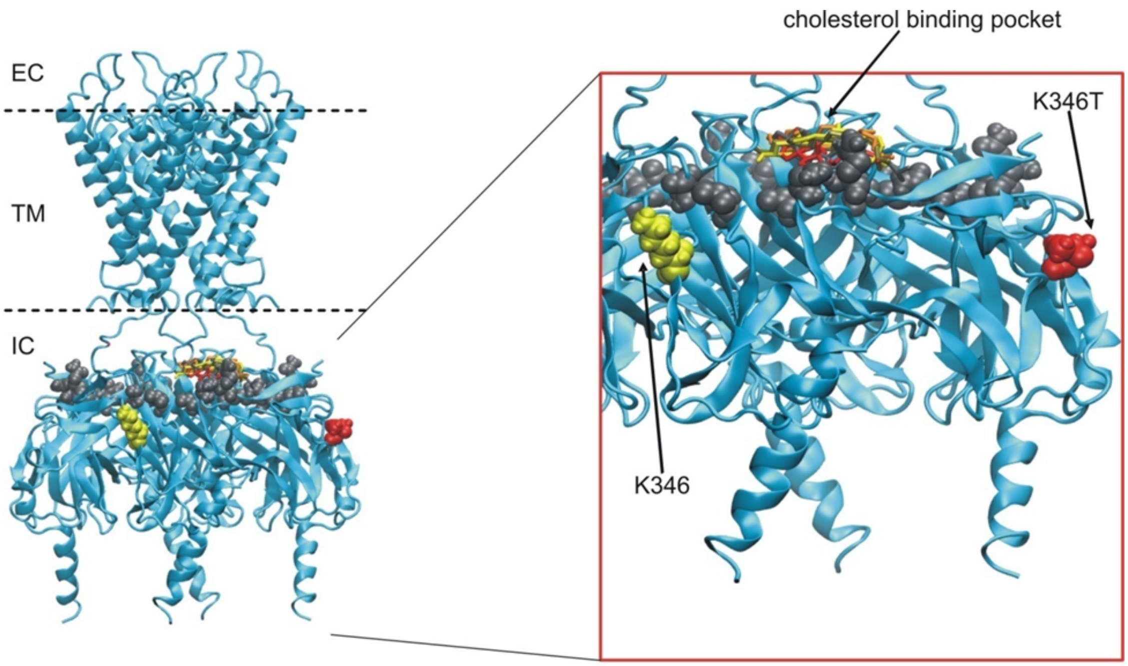

Supplementary figure S5

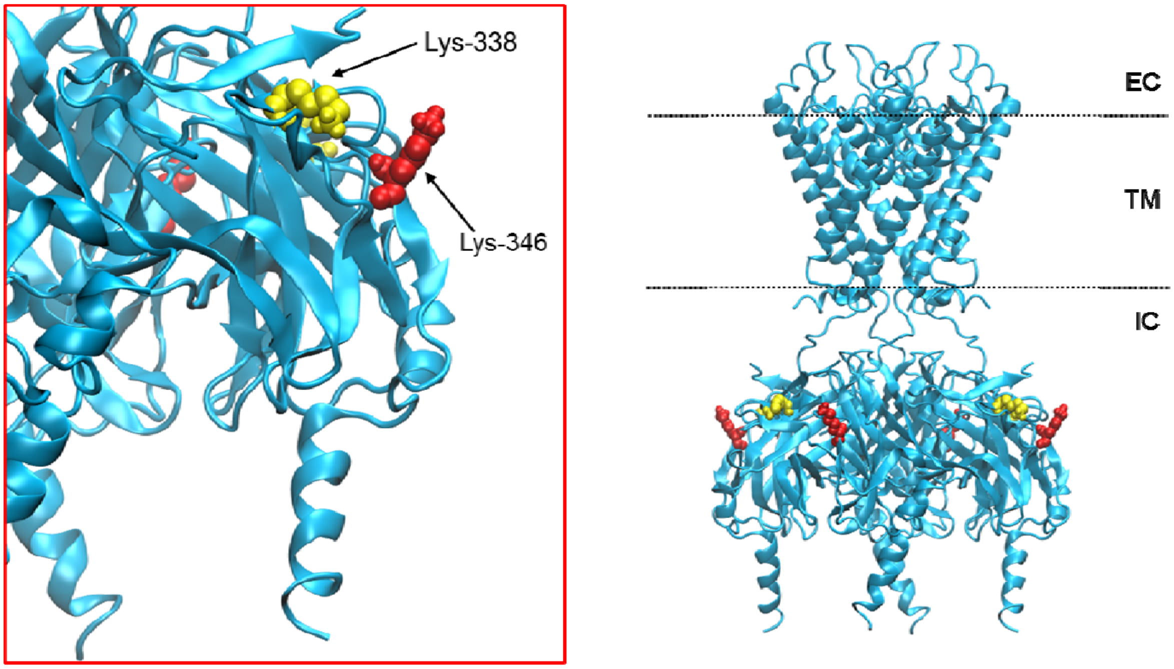

Supplementary figure S6
